# Evaluation of a competency based medical curriculum in a Sub-Saharan African medical school

**DOI:** 10.1186/s12909-022-03781-1

**Published:** 2022-10-14

**Authors:** Jane McKenzie-White, Aloysius G. Mubuuke, Sara Westergaard, Ian G Munabi, Robert C Bollinger, Robert Opoka, Scovia N Mbalinda, David Katete, Yukari C Manabe, Sarah Kiguli

**Affiliations:** 1 Division of Infectious Diseases, School of Medicine, Johns Hopkins, Baltimore, USA; 2 College of Health Sciences, Makerere University, P.O. Box 7072, Kampala, Uganda

**Keywords:** Competency based Medical Education, Evaluation, Makerere University

## Abstract

**Background:**

Medical schools in Sub-Saharan Africa have adopted competency based medical education (CBME) to improve the quality of graduates trained. In 2015, Makerere University College of Health Sciences (MaKCHS) implemented CBME for the Bachelor of Medicine and Bachelor of Surgery (MBChB) programme in order to produce doctors with the required attributes to address community health needs. However, no formal evaluation of the curriculum has been conducted to determine whether all established competencies are being assessed.

**Objective:**

To evaluate whether assessment methods within the MBChB curriculum address the stated competencies.

**Methods:**

The evaluation adopted a cross-sectional study design in which the MBChB curriculum was evaluated using an *Essential Course Evidence Form* (ECEF) that was developed to collect information about each assessment used for each course. Information was collected on: (1) Assessment title, (2) Description, (3) Competency domain (4) Sub-competency addressed, (5) Student instructions, and (6) Grading method/details. Data were entered into a structured Access data base. In addition, face-to-face interviews were conducted with faculty course coordinators.

**Results:**

The MBChB curriculum consisted of 62 courses over 5 years, focusing on preclinical skills in years 1–2 and clinical skills in years 3–5. Fifty-nine competencies were identified and aggregated into 9 domains. Fifty-eight competencies were assessed at least one time in the curriculum. Faculty cited limited training in assessment as well as large student numbers as hindrances to designing robust assessments for the competencies.

**Conclusion:**

CBME was successfully implemented evidenced by all but one of the 59 competencies within the nine domains established being assessed within the MBChB curriculum at MaKCHS. Faculty interviewed were largely aware of it, however indicated the need for more training in competency-based assessment to improve the implementation of CBME.

**Supplementary Information:**

The online version contains supplementary material available at 10.1186/s12909-022-03781-1.

## Background

Medical education has witnessed a paradigm shift towards competency-based medical education (CBME) over the past two decades. CBME is a concept where teaching, learning and assessment are driven by the needs of the population which in turn direct the kind of competencies learners should attain in order to address those needs [1]. Therefore, the goal of CBME is to graduate health professionals with defined abilities, who can meet local health care demands and optimize their care of the communities they serve [2, 3].

CBME curricula use assessments to verify that students have acquired each of the established competencies and to provide opportunity for progressive monitoring, mastery and maintenance of skills throughout the educational process [4, 5]. These assessments can utilize multiple methodologies, including written and oral exams, defined problem sets, presentations of research, as well as observations of student practice and behavior in the classroom, clinic or field through the use of the Objective Structured Clinical Examinations (OSCEs) and student portfolios [6]. The specific assessment methods chosen should have the psychometric rigor to demonstrate that the students have successfully attained the targeted competencies and have reached an acceptable level of proficiency [6]. For instance, assessment methods may differ between the pre-clinical and clinical years or when transitioning from knowledge-based to performance-based assessments [7]. Since all assessment methods have limitations, ideally competencies should also be addressed by multiple assessment methods [8]. The ability to design robust, valid and reliable assessment methods is a cornerstone of CBME. In addition, faculty must be supportive of utilizing optimal assessment methods and should have the knowledge and skills to develop valid assessments [3], if implementation of CBME curricula is to be effective and sustainable [9].

Therefore, any educational institution utilizing CBME should perform iterative systematic evaluations of their competency assessments and assess the willingness and ability of faculty to optimize their course assessments [10–13]. While implementation of competency-based reforms has been written about in other contexts, little is published on the implementation and evaluation of CBME in sub-Saharan Africa medical schools [2, 12].

The goal of the study was to evaluate the CBME curriculum in a sub-Saharan Africa medical school, Makerere University College of Health Sciences (MaKCHS), to evaluate whether the assessment tools within the current curriculum address the stated competencies to better inform future curriculum changes. In this paper, we report the method and findings from this evaluation.

## Methods

### Design and setting

In 2015, MaKCHS transitioned to a CBME curriculum for its Bachelor of Medicine and Bachelor of Surgery (MBChB) programme to improve the quality of professionals trained who would be able to effectively address the prevailing health needs of the population. The MBChB programme spans five years and involves two phases namely: the pre-clinical phase (1st and 2nd years) and the clinical phase (3rd, 4th and 5th years). (Supplemental Table 1) After 5th year, successful students then proceed to do a mandatory internship period of one year before they can get registered and practice as independent professionals.

In 2018, MaKCHS (and partner institutions) received an NIH grant, Health Professional Education Partnership Initiative (HEPI) Project, funded by the National Institutes of Health, with a goal of improving service delivery of the Ugandan health system through strengthened interdisciplinary health professional education and research training to produce graduates with competencies to address the priority health needs of the population. One of the key areas addressed by the HEPI project was the implementation of competency-based education in the partner institutions in Uganda. Therefore, a HEPI study team was formed and included individuals from MaKCHS and Johns Hopkins University. The team undertook a systematic review of all assessments conducted throughout the MBChB curriculum. Since all competencies established for a curriculum are considered necessary skills the students must have, one parameter of success for a CBME curriculum would be when all established competencies are in fact assessed within the curriculum.

This was a cross-sectional study conducted at MaKCHS in which assessment tools used in the MBChB curriculum were evaluated and mapped to the stated competencies. In addition, individual interviews were conducted with faculty who teach the various courses in the curriculum to obtain further understanding of the assessment of the stated competencies. This enabled us to better triangulate the findings from the curriculum with responses from faculty.

### Data collection and analyses

A structured *Essential Course Evidence Form* (ECEF) was developed by the HEPI study team to collect information about each assessment used for each course in the MBChB program. The ECEF included basic course information (course title, number, and instructors), as well as instructions for the Course Coordinator to answer six questions to provide specific details for each of the assessments they use for their course. The 6 questions included: (1) Assessment title, (2) Description of assessment format, timing, purpose, (3) Competency domain (4) Sub-competency addressed, (5) Student instructions to complete the assessment, and (6) Grading method/details. A final optional, open-ended question provided faculty an opportunity to share opinions, challenges and suggestions for improvements to their assessment(s).

MaKCHS administration provided the HEPI study team with a list of competencies and courses in the MBChB curriculum. A list of names and e-mail addresses of the Faculty Course Coordinators were also provided. For every course, there were one to two coordinators listed.

A mixed methods approach was utilized to complete the ECEF for each assessment used for each course. The process included three steps: an email sent to faculty Course Coordinators, a face-to-face interview with Course Coordinators and a review of course materials by the HEPI study team. All data collected on the ECEF were entered in a Microsoft Access® database.

**Step 1 - Email**. From February – November 2019, the HEPI study team emailed all the Course Coordinators to participate in this exercise. The email explained the purpose and process of the curriculum evaluation and a request for them to complete the attached ECEF. A list of MBChB curriculum competencies was also attached.

**Step 2 - Interviews**. For Course Coordinators unable to complete the ECEF by email, the HEPI study team also conducted in-person interviews with them between January 13–25, 2020, at Makerere College of Health Sciences. Interviews were scheduled for one hour and faculty coordinators were invited to bring any additional faculty they felt could contribute to the discussion. During each interview, the HEPI study team asked faculty each question on the ECEF form and collected information on each assessment to complete the form. Additional clarifying questions were asked if needed. Responses to all six questions were documented for each assessment on the ECEF, as well as the faculty comments in response to the final question. In-person interviews were done by 2 members of the HEPI study team (JMW and SW).

During the interviews many faculty course coordinators were unclear about which competencies were addressed for each assessment. Assessments were developed by teaching faculty based on the course content. Faculty were also asked to provide hard or digital examples/copies of their course assessments. These documents included course exams (written, oral, and practical), tutorial and seminar questions, logbooks, report requirements, and clinical and laboratory checklists. Scans and digital copies of assessments were stored on a secure Johns Hopkins University server and accessible only to the HEPI study team conducting the analysis.

**Step 3 - Analysis**. These materials were reviewed by 2 members of the HEPI study team (JMW and SW) to confirm and complete the ECEF. Using a face validity approach, each reviewer independently looked at specific items within the assessment, e.g., test questions, check list items, performed skills, etc., to determine if a competency domain was being addressed and if so, which subcompetencies within that domain. Discrepancies between the reviewers were discussed until consensus was achieved. If ECEF data were not available from the email solicitation, the interview or provided course materials, assessments were imputed when possible from a similar course within the same department, or from a similar assessment within the same course. After completion of the ECEF for each assessment and each course, the data were entered into a structured Microsoft Access® database, by a member of the HEPI study team.

### Mapping of required competencies to course assessments

A *Curriculum Matrix (CM)* in Microsoft Excel® was developed to determine whether all competencies were being assessed. The CM listed the curriculum competencies down the first column and the courses across the top row. When an assessment within a course was determined to address a competency, it was documented on the CM at the intersection of that competency and course. An ‘X’ at the intersection of a course and competency indicates the competency was addressed at least one time by an assessment within that course but cannot distinguish whether other assessments within the course also addressed the same competency. The Microsoft Access® database, however, did provide information on the number of times each competency was addressed, both within a course and across the curriculum. Each individual assessment and the competencies each addressed were entered in the database, capturing data on the number of times a competency is addressed, both within a course and across the curriculum. In cases where the assessment was known but supporting information was not available to determine competencies addressed by that assessment, the assessment was recorded in the database, however, no competencies were mapped to that assessment.

### Classification of assessment methods

For purposes of analysis, the HEPI study team established a set of categories by which all documented assessments could be assigned. Characteristics of each assessment were reviewed, e.g., format, context, skills addressed. Using these assessment characteristics, categories for the MBChB curriculum were defined based on similarities in assessment format and student output expected. Each assessment documented on the ECEF was assigned by the HEPI study team to one of these method categories.

### Documenting faculty feedback

All faculty participating in the in-person interviews were provided an opportunity to respond to the final, optional question on the ECEF form. Each were asked what improvements they felt would be beneficial in improving assessment capabilities, what challenges they experienced, and what suggestions they had for future implementation of the CBME at Makerere. The HEPI study team asked follow-up questions, as needed, for purposes of clarification. All comments were recorded on the ECEF form and added to the database. All faculty responses were anonymously aggregated. Using a thematic analysis approach, recurring major themes were identified.

### Ethical considerations

Approval to conduct this study was granted by the School of Medicine Research Ethics Committee, Makerere University (REC No. 2019-007). Informed consent was obtained from faculty to participate in interviews. Confidentiality of the participant responses was ensured. All methods involved in data collection were performed according to the regulations and guidelines of the Declaration of Helsinki.

## Results

### Program courses and competencies

The MBChB curriculum was obtained from the administrative office of MaKCHS in early 2019 and consisted of 62 courses over 5 years, focusing on preclinical skills in years 1–2 and clinical skills in years 3–5. Fifty-nine competencies were identified and classified by MaKCHS into the nine domains.

Of the sixty-two courses, the names and contact information of faculty coordinators were provided for sixty-one. Emails to the faculty yielded partial ECEF responses for only twelve courses (20%). Therefore, 30 in-person interviews with 39 faculty course coordinators conducted by 2 members of the HEPI study team (JMW and SW) held on-site at MaKCHS were the primary source of data collected to complete ECEFs for fifty-seven courses (92%). Some faculty were coordinators for multiple courses and were interviewed about each. Data were not available for five (8%) of the sixty-two courses, due to lack of faculty contact (n = 1) or inability to schedule an interview (n = 4). These courses were: (a) *Ethics, First Aid & Professionalism*, (b) *Introduction to Public Health – Community Based Education, Research and Service (COBERS)*, (c) *Health Systems, Management and Leadership – COBERS*, d) *Forensic Medicine* and e) *ENT (Ear, Nose & Throat)*.

### Determination of competencies assessed

A total of 188 assessments were documented on the ECEFs for 57 courses. Due to insufficient detail provided, competencies were not able to be assigned for 11 assessments documented on the ECEF of three courses: (a) Systemic Pathology I (Gastrointestinal tract, Cardiovascular system, Respiratory system & Central Nervous System); (b) Systemic Pathology II (Endocrine, Central Nervous System, Genitourinary tract); & (c) Chemotherapy. The remaining 177 assessments from fifty-four courses documented that 58 (98%) of the 59 competencies were assessed at least once in the curriculum. One competency in Domain 8-Continuous Improvement of Care Through Reflective Practice (8.ii *Demonstrate lifelong learning in the areas of science, health care and public health*) was not addressed in any of the 177 assessments reviewed. Nine (15%) of the 59 competencies were addressed by less than five assessments. These competencies fall within domains 3-Critical Inquiry and Scientific Method (1 of 4 competencies), 6-Leadership and Management (4 of 7 competencies), 7-Population Health Skills (2 of 6 competencies), 8-Continuous Improvement of Care Through Reflective Practice (2 of 7 competencies). (Supplemental Table 2)

**Fig. 1:**
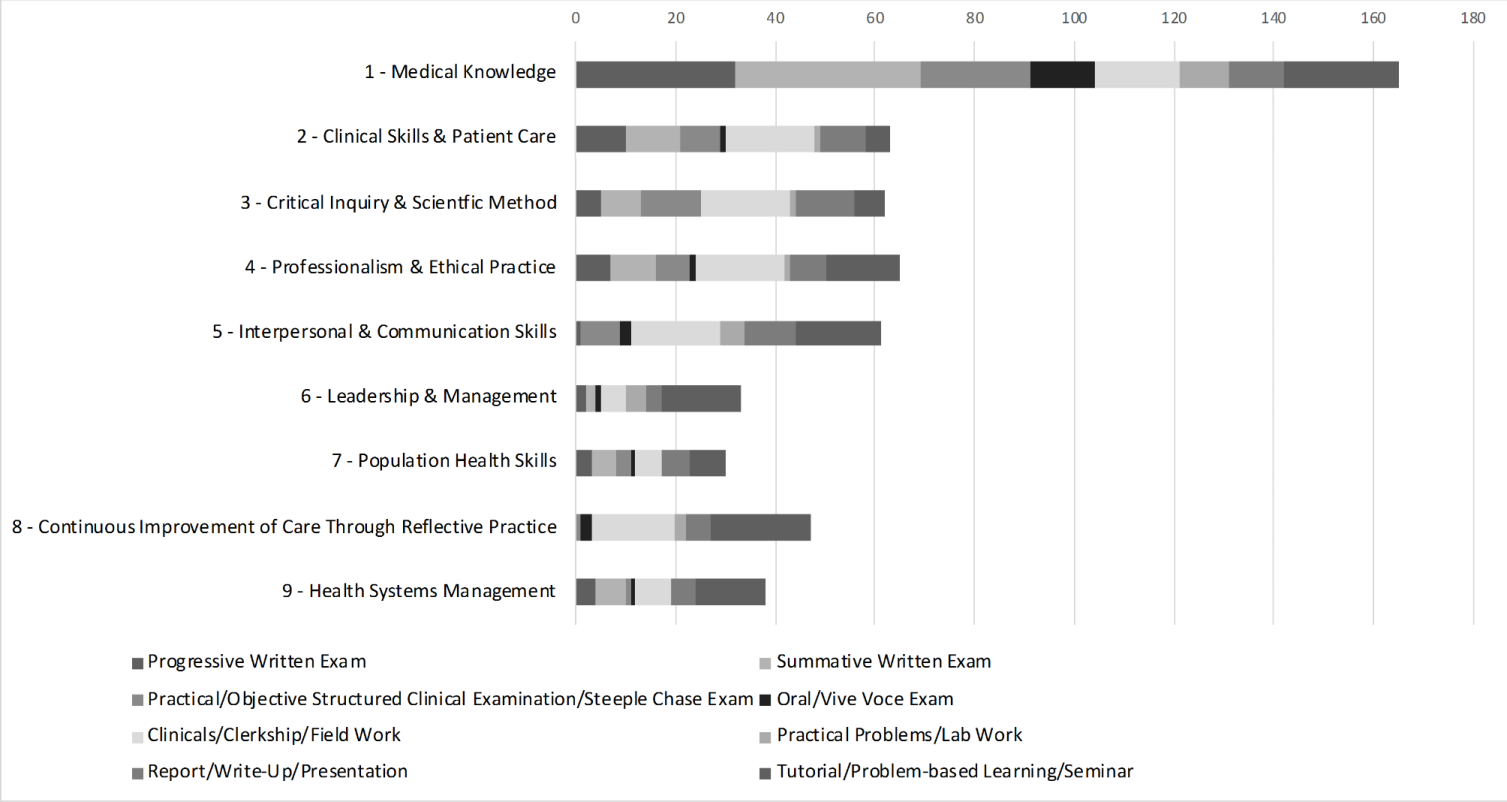
Number of Times Each Method is Used for Each Competency

A single course could have multiple assessments that address the same competency. Therefore, we also looked at the number of courses across the curriculum that provided an assessment for each competency. Fourteen (24%) of the fifty-nine curriculum competencies were addressed by fewer than five courses across the curriculum. Figure 1 shows the number of times each competency domain is addressed and which assessment method was used for each. This graph highlights that Domain 1-Medical Knowledge is assessed using all assessment method categories and is the most heavily assessed of all competency domains, addressed in 165 (88%) of the 188 total assessments documented. Figure 1 also shows that all domains are addressed by a variety of assessment methods and each is addressed by a minimum of 30 assessments.

### Determination of assessment methods

To determine the frequency that each assessment method is used across the MBChB curriculum and the alignment between assessment method and competency, we looked at the method of assessment frequency and the number of times each method was used throughout the MBChB curriculum. As described in Table 1, a method category was assigned to each of the 188 assessments documented for 57 courses. While both written exams mainly differ in the amount of course content addressed, progressive (given mid semester) and summative (given end of semester) exams were used with high frequency and as such, we chose to categorize them separately. Also shown in Table 1 is the usage of each assessment method, both as an absolute number of courses the method was used and as a percentage of the total number of courses in the curriculum. Each method is used in a minimum of 20% of the courses within the MBChB curriculum. Progressive and summative written exams are the most heavily used assessment method (65% & 77% respectively) across courses in the curriculum, while practical problems/lab work and reports/write-up/presentation are used in 21% and 23% of courses, respectively.

**Table 1:** Assessment Method Category and Description and Number of Courses Using Each

Category	Description	# Courses	% of Total Courses
**Progressive Written Exam**	a written exam given sometime during a course	37	65
**Summative Written Exam**	a written exam given at the end of a course/semester	44	77
**Practical/Objective Structured Clinical Examination/Steeple Chase Exam**	includes any form of practical performance or hands-on application/demonstration of skill	23	40
**Oral/Vive Voce Exam**	includes all forms of oral examination	15	26
**Clinicals/Clerkship/Field Work**	includes all clinical work on the wards, community, and field work	18	32
**Practical Problems/Lab Work**	any assignment requiring students to respond/complete in specific problems, questions, or laboratory work	12	21
**Report/Write-Up/Presentation**	any assignment requiring students to generate a report or present on a topic they’ve explored, e.g., journal club, or project they’ve done.	13	23
**Tutorial/Problem-based Learning/Seminars**	any activity requiring students to participate in a topical discussion or individual/group project they’ve been assigned and/or asked to research. Students could be required to share with larger group or participate in discussion with faculty.	26	46

**Fig. 2:**
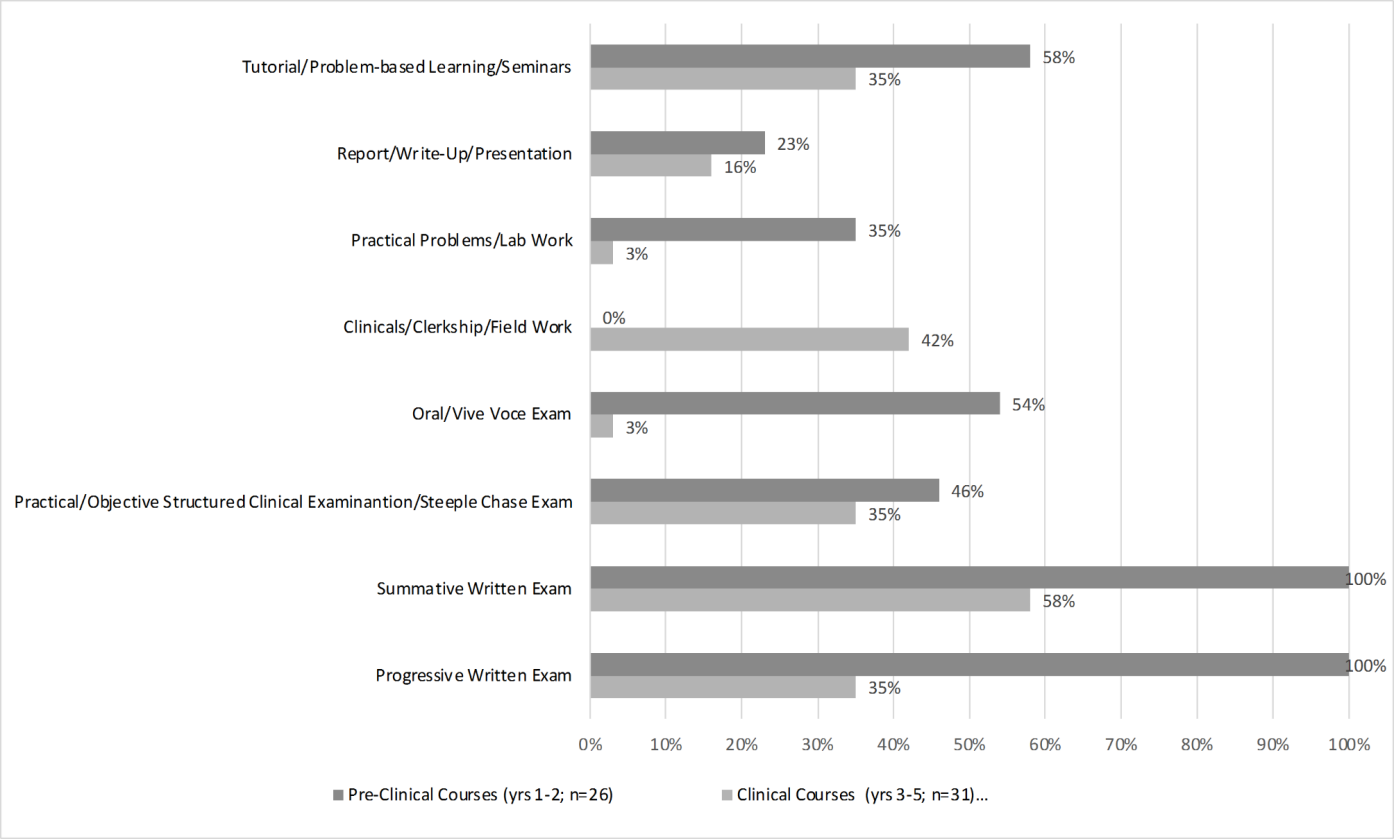
Percent of Courses in the Pre-Clinical and Clinical Years Using Each Assessment Category

As shown in Fig. 2, all of the 26 pre-clinical courses (100%) used both a progressive and summative exam, compared to their use in the 31 clinical courses, 35% and 58% respectively.

### Faculty feedback regarding the course assessments

The responses to the final question on the ECEF form *(What improvements would be beneficial in improving assessment capabilities, what challenges and suggestions do you have for future implementation of CBME at Makerere?)* were aggregated and from these responses, two major themes were identified.

1. **Faculty Training**: Faculty reported a lack of available training in instructional, assessment, and evaluation strategies for a CBME curriculum. Responses indicated training in these areas would likely lead to improved consistency in assessment across all faculty.
2. **Insufficient teacher/student ratio**: From the responses, it was observed that the faculty numbers did not keep pace with the increasing number of students and has affected some of the assessments. This low teacher/student ratio was reported to have an impact on faculty ability to provide adequate assessment materials and sufficient teaching and learning space to manage student numbers. This limited the ability to use some assessment methods and to conduct learning and assessment activities (such as practicals/hands on work, problem-based learning, small projects, oral exams, OSCEs, written examinations). Insufficient teacher/student ratios also impacted faculty ability and time to deliver valuable feedback to all students after the assessments.

## Discussion

Makerere University College of Health Science’s transition to CBME was designed to optimize skills development amongst health professionals that would ultimately be needed to address Uganda’s priority health needs [1]. As reported in previously published work describing this change process, nine key competency domains were defined along with their respective sub-competencies [1]. The medical curriculum was subsequently revised to incorporate these new competencies, but also to align the teaching, learning and assessment methods with the defined competencies. However, since the transition to a CBME curriculum, no formal evaluation had been conducted to determine whether assessments being performed did in fact address the defined competencies. With the goal of CBME to provide students the skills to optimize health care of local communities served, it would be expected that all established competencies are assessed and that assessment takes place multiple times during the curriculum using multiple methods. This study was done to provide a monitoring and evaluation framework to map course assessments to established competencies for the MBChB curriculum. The methodology and outcome of such evaluations can inform curriculum improvement and competency adjustments and provide an iterative process to ensure the MBChB curriculum at Makerere University College of Health Sciences continues to be aligned with the health care demands of the Ugandan population.

Results from the evaluation indicated that all nine competency domains and 98% of the 59 competencies established for the MBChB program were being addressed by assessments utilized throughout the curriculum. However, competencies across several domains were assessed less than five times across the curriculum. Although five times was arbitrarily chosen, this raises the questions whether these competencies are being adequately assessed and whether they remain relevant within the curriculum. If these competencies are still relevant, efforts to further incorporate these into the current curriculum teaching and assessment framework are needed. However, it should be noted that from the evaluation, all but one of the fifty-nine competencies were targeted by at least one assessment method which is a positive finding. With shifting local health care demands, faculty changes, and assessment modifications, this highlights the need to continually evaluate established competencies for a curriculum and the assessments that address them. In doing so, competencies and assessments can be revised to ensure the curriculum continues to optimize the training students receive to care for the communities they serve.

In addition to knowing if competencies are being addressed in course assessments, it is helpful to understand how they are being addressed. All assessment methods have limitations and as such, the adoption of multiple assessment methods across a curriculum provide opportunity to align method to the competency. Results of this evaluation demonstrate that the courses across the MBChB curriculum use a range of assessment methods to assess program competencies, ranging from written exam to problem-based learning, use of OSCEs, practicals etc. Each of the assessment methods is used in over 20% of courses evaluated within the curriculum as well as in assessing all nine competency domains.

Differences in the application of the various assessment methods in the pre-clinical versus clinical years were also found. Although these differences might be expected, especially in the use of written and oral exams, both methods are used significantly less frequently in the clinical years compared to pre-clinical years, where competencies are focused more on psychomotor skills necessary for patient care.

As with any change in curriculum, it is the faculty that ultimately carry out these changes. Results of this evaluation demonstrate that within their courses, faculty have been using assessments that do, overall, address the competencies identified for the MBChB program. However, this evaluation also provided important feedback both to Makerere leadership and its faculty. Despite the overall success in addressing the competencies for the MBChB program, instructional training and insufficient faculty/student ratios are two key areas faculty identify as impacting their ability to successfully implement a CBME curriculum at Makerere. The first area underscores the significance of continued faculty development to not only equip the teaching faculty with knowledge of the curriculum competencies but also to provide ongoing training of the necessary skills to successfully teach and assess these competencies. Indeed, in a context where CBME is a new concept to teaching faculty, faculty expressed a need for greater understanding of the concept itself, as well as training in varied assessment methods. This requires continued efforts to train faculty in the most feasible and valid assessment methods for the various competencies amidst increasing student numbers. This observation is likely generalizable to other institutions struggling with the implementation of CBME especially from low- and middle-income countries (LMIC) and where student numbers are increasing throughout sub-Saharan Africa, as has been previously reported in [14].

Addressing the second area, insufficient faculty/student ratios, highlights a potential burden on faculty in carrying out assessment methods that require higher levels of individual feedback. This also has the potential to lead to inconsistencies across faculty in terms of the quality and quantity of feedback provided. Addressing this area of faculty concern is beyond the scope of this evaluation, however, it does highlight a critical issue in many LMIC medical schools and most especially the medical schools in Sub-Saharan Africa that were mandated during the implementation of the Medical Education Partnership Initiative (MEPI) programme to increase the number doctors graduating [14].

The complexity of implementing and evaluating a CBME curriculum has been reported by groups outside SSA [15–19]. These primarily have focused on post-graduate training [15–18], using national assessment data and promotional timelines [15], survey of competence committees [17] and lecture and syllabus review with student feedback [19]. However, the goal of this study was to specifically assess alignment of student assessments throughout the curriculum to established programme competencies. It is not possible to compare these findings with other schools in sub-Saharan Africa since there is a dearth of published research on the mapping of assessments to established competencies in CBME curriculum.

This evaluation not only provides information to MBChB teaching faculty and leadership on alignment of curriculum competencies and assessment, but also provides a framework for conducting such evaluations for other Sub-Saharan Africa medical schools implementing CBME. Globally, many medical schools are implementing CBME and many, especially from LMICs, are faced with the same questions addressed in this evaluation. This study thus provides some useful insights into implementing a CBME curriculum, essentially through an ongoing, iterative review of defined competencies and corresponding assessment as well continued faculty training in CBME strategies.

## Limitations and challenges with the review

We interfaced with faculty who lead the courses for the interviews, but not all teaching faculty in the institution which may have led to the omission of some information. In the data capture, we collected only formal assessments from documents and from faculty interviewed. We did not capture informal assessments with no standardized performance measure, all of which could certainly play a role in addressing the stated competencies. Data was also collected as a snapshot and such cross-sectional data may not capture variations over time that could influence outcomes. Finally, the eight method categories defined were subjective and assignment of all assessments to these categories for analysis were at the discretion of the HEPI study team doing the analysis.

## Conclusion and recommendations

CBME was successfully implemented at MaKCHS as evidenced by all but one of the fifty-nine competencies within the nine domains established for the MBChB program being assessed within the curriculum and by the use of multiple methods. The faculty interviewed were largely aware of it. We recommend more faculty training in CBME to align the competencies with the assessment methods within a formal standardized framework across the pre-clinical and clinical years and across departments. We also recommend future longitudinal studies to further interrogate the curriculum over some time across the years, to involve more faculty and other key stakeholders like the community and graduates of the programme to assess whether the curriculum is addressing the community’s health needs.

## Electronic supplementary material

Below is the link to the electronic supplementary material.

Supplementary Material 1

Supplementary Material 2

## Data Availability

The datasets used and/or analysed during the current study are available from the corresponding author on reasonable request.

## Abbreviations

MaKCHS: Makerere University College of Health Sciences.
CBME: Competency Based Medical Education.
HEPI: Health Professions Education Partnership Initiative.
ECEF: Essential Course Evidence Form.
CM: Curriculum Matrix.
